# Effects of an exercise intervention on maternal depression, anxiety, and fatigue: a systematic review and meta-analysis

**DOI:** 10.3389/fpsyg.2024.1473710

**Published:** 2024-11-18

**Authors:** Haoran Yu, Qinglei Mu, Xunjin Lv, Shuainan Chen, Hao He

**Affiliations:** ^1^Institute of Sports Training, Chengdu Sport University, Chengdu, Sichuan, China; ^2^Institute of Physical Education, Chengdu University, Chengdu, Sichuan, China; ^3^Institute of Physical Education, Jiangxi Normal University, Nanchang, Jiangxi, China; ^4^Institute of Physical Education, Northeast Normal University, Changchun, Jilin, China

**Keywords:** exercise intervention, maternal, depression, anxiety, fatigue, meta-analysis

## Abstract

**Background:**

Existing meta-analyses suggest that exercise intervention may play a crucial therapeutic role in improving maternal depression, anxiety and fatigue symptoms. However, the efficacy varies across different exercise content, duration, frequency, cycle, intensity, format and intervention period.

**Objective:**

Using meta-analysis to propose the best intervention program and examine the effect of exercise intervention on maternal depression, anxiety, and fatigue.

**Methods:**

Five databases (PubMed, Web of Science, Embase, Cochrane Library, CNKI) were searched from inception to June 2024, a total of 37 literatures were included. The methodological quality of the included literatures was assessed using the Cochrane Risk of Bias tool and the PEDro scale. When heterogeneity was high, we used random-effects models. Funnel plots were used to assess publication bias. Sensitivity analysis was used to verify the robustness of the combined results. Subgroup analysis was used to explore sources of heterogeneity.

**Results:**

Exercise has beneficial effects on the improvement of maternal depression [*g* = −0.71, 95%CI (−0.93, −0.49), *p* = 0.00], anxiety [*g* = −1.09, 95%CI (−1.42, −0.76), *p* = 0.00] and fatigue [*g* = −0.64, 95%CI (−0.88, −0.40), *p* = 0.00] symptoms. Postnatal interventions may be more effective than prenatal. Low-moderate intensity yoga with group + individual, 4–5 times/week, 40–60 min/time, duration 4–8 weeks is most effective in improving depressive symptoms. Low-intensity yoga with group + individual, 4–5 times/week, 40–60 min/time, duration 4–8 weeks is most effective for improving anxiety symptoms. Low-intensity Pilates with group, 1–2 times/week, 40–60 min/time, duration 4–8 weeks is most effective for improving fatigue symptoms.

**Conclusion:**

This meta-analysis demonstrates the positive effect of exercise on improving maternal depression, anxiety and fatigue and suggests the best intervention program. Maternal perceptions that postpartum exercise is safer may account for the better outcomes of postpartum intervention. Further higher quality and large-scale trials are needed to substantiate our findings.

**Systematic review registration:**

https://www.crd.york.ac.uk/PROSPERO/, CRD42024567987.

## 1 Introduction

Maternal, including pregnant and postpartum women, experience significant physical, emotional, and financial changes during pregnancy and childbirth. During this period, women are more susceptible to depression and anxiety compared to other life stages (Hunter et al., 2024). It may also one of the most fatiguing phases of a woman’s life (Wilson et al., 2019). Globally, the prevalence of prenatal and postpartum depression ranges from 10 to 33.3% (Ji et al., 2024); prenatal and postpartum anxiety ranges from 16 to 25% (Glasheen et al., 2010; Field, 2017) and maternal fatigue is as high as 41–90% (Nazik and Eryilmaz, 2014; Reeves et al., 1991). The high prevalence of maternal depression, anxiety, and fatigue significantly affects maternal physical and mental health and quality of life, even causing harm to the fetus or baby (Dunkel Schetter and Tanner, 2012), e.g., low birth weight (de Bruijn et al., 2009), developmental delay and preterm labor (Field, 2010). Compared to non-depressed, anxious maternal, depressed, anxious maternal are more likely to be fatigued (Taylor and Johnson, 2013), have a greater fear of childbirth (Hall et al., 2009), and their newborns are at higher risk for depression and anxiety during childhood and adolescence (Kingston et al., 2012). Although medication is an important treatment for depression and anxiety, it is important to consider the adverse effects of antidepressant and anxiolytic medications, e.g., may cause congenital disability in newborns and maternal headache and drowsiness (Latendresse et al., 2017). Evidence suggests that fatigue can lead to decreased maternal physical and mental work capacity and even feel helpless, seriously affecting maternal and baby health and life satisfaction (Liu et al., 2020), and may exacerbate depression and anxiety symptoms (Dennis and Ross, 2005).

Exercise, as a safe and practical non-pharmacological intervention, is important for treating maternal depression, anxiety and fatigue (Cleare et al., 2015). The results of a meta-analysis showed that after exercise intervention, significant and overall improvements in maternal depression and anxiety symptoms were observed (Liu et al., 2022). This may be due to the fact that appropriate exercise can regulate the maternal endocrine system, activate potential targets in the brain, plus the concentration of monoamine neurotransmitters in the brain, to achieve the objective of improving the depression and anxiety symptoms (Gujral et al., 2017; Nascimento et al., 2015). Increasing prenatal weight, postpartum debilitating mobility and physical inactivity are important causes of maternal fatigue (Badr and Zauszniewski, 2017; Liu et al., 2024). Research has confirmed that exercise interventions can improve maternal self-care, daily activity and sleep quality, and alleviate low back pain, all of which can help improve physical fatigue (Jia et al., 2021). Furthermore, exercise can alleviate bad moods, reduce maternal body image worries, which helps to improve psychological fatigue (Nascimento et al., 2014). Currently, exercise intervention is widely encouraged by virtue of its advantages of convenience, economy and side effects. However, the effects of different exercise content, duration, frequency, cycle, format, intensity and intervention period remain unclear. As more women exercise around pregnancy to improve physical and mental health and relieve fatigue, researchers and clinicians need to identify and implement best practices in treatment. Based on this, the primary aim of this study is to investigate the effects of different exercise content, duration, frequency, cycle, format, intensity, and intervention period on the overall intervention effect, and to propose the best intervention program. Analysis the effects of exercise interventions on maternal depression, anxiety, and fatigue. To provide evidence and references for the development of more targeted and comprehensive exercise intervention programs.

## 2 Methods

The study followed the PRISMA guidelines and the Cochrane Handbook for meta-analysis and systematic review (Page et al., 2021; Higgins et al., 2019). The research was registered on the International Prospective Register of Systematic Reviews (PROSPERO), identifier: CRD42024567987. Conducted a self-review based on the PRISMA 2020 Checklist (Supplementary material).

### 2.1 Inclusion and exclusion criteria

According to the PICOS principles, literature inclusion criteria included: (1) Participants: maternal, age ≥ 18, without pregnancy complications or other illnesses. (2) Interventions: the intervention group performed exercise (yoga, aerobic exercise, etc.). (3) Controls: the control group received non-exercise (usual care, daily activities, etc.). (4) Outcomes: depression, anxiety and fatigue rating scales, results presented as mean (M) ± standard deviation (SD). (5) Types of studies: randomized controlled trials (RCT).

Exclusion criteria: Studies involving (1) maternal participants with gestational diabetes, high blood pressure, other diseases, or severe mental disorders requiring pharmacological intervention. (2) non-RCT, conference abstracts, and review articles. (3) Studies with incomplete data reporting. (4) Non-Chinese and non-English literature. (5) Animal studies.

### 2.2 Literature search

Search in PubMed, Web of Science, Embase, Cochrane Library, and CNKI databases from inception to June 2024.The search strategy was based on medical subject headings (MeSH) and free words with “AND” and “OR” linking, e.g.: (“Pregnancy [Mesh]” OR “Postpartum Period [Mesh]” OR “maternal” OR “pregnant” OR “post-pregnancy” OR “Postpartum Period”) AND (“Exercise [Mesh]” OR “Exercise interventions” OR “physical exercise” OR “sport” OR “physical activity” OR “exercise” OR “yoga”) AND (“Depression [Mesh]” OR “Depression, Postpartum” OR “Anxiety [Mesh]” OR “Fatigue [Mesh]” OR “Burnout” OR “tired”). A subsequent supplement was conducted to trace relevant systematic reviews and references of included papers for those not having been retrieved. The complete search strategy is in Supplementary material.

### 2.3 Literature screening

The retrieved literature was imported into Endnote X9.1, after removing duplicates, two researchers independently screened the titles and abstracts according to the inclusion and exclusion criteria, and then read the full texts for further screening. If the results were consistent, the literature was included in this study, if not, it will be discussed with the 3rd researcher until a consensus was reached.

### 2.4 Data extraction and coding strategy

Two researchers independently extracted data from the eligible literature using an agreed form, 98.35% concordance of data extracted by two researchers. Disagreements were discussed with the 3rd researcher until consensus was reached. The main components extracted were: (1) basic information about the included literature (first author, publication year, country); (2) subject characteristics (sample size, pregnancy week, other characteristics); (3) intervention in the experimental group (exercise content, single exercise time, frequency, exercise cycle, exercise form, intensity, intervention period); (4) intervention in the control group; and (5) measure tools.

Exercise content was coded as: yoga, Combination of aerobic exercise (including aerobic-based walking, dance, etc.) and Pilates. Single exercise time was coded as: 10–30 min/time, 40–60 min/time and 75–90 min/time. Exercise frequency was coded as: 1–2 times/week, 3 times/week and 4–5 times/week. Exercise cycle was coded as: 4–8 weeks, 9–12 weeks and 14–16 weeks. Exercise intensity was coded as: low, medium, low-medium and medium-high. Exercise format was coded as: group, individual and group + individual. Intervention period was coded as: prenatal, postpartum and prenatal+ postpartum.

### 2.5 Quality assessment

The methodological quality of the literature was evaluated using the Cochrane Risk Assessment Tool, which includes seven items: Random sequence generation, allocation concealment, blinding of participants and personnel, Blinding of outcome assessment, incomplete outcome data, selective reporting, other bias. Each literature was assessed in three options: high risk, unclear risk, and low risk. Quality risk assessment of the included literature was performed using the PEDro scale (<4: low quality, 4–5: medium quality, 6–8: high quality, and 9–10: very high quality). It was independently by two researchers, and when results were inconsistent, it was resolved by discussion with the 3rd researcher until a consensus was reached. In addition, we assessed the evidence level of the included literature according to the GRADE system (Supplementary material).

### 2.6 Statistical analysis

Review Manager 5.4 was used for the methodological quality assessment of the literature, and Stata17 was used for publication bias test (including Egger’s test and Begg’s test), sensitivity analyses, combining effect sizes, forest plotting, and subgroup analyses. The data used in this study are the change values of M and SD from baseline to endpoint. If it cannot be extracted directly, it is estimated according to the following formula: *M* = *M*_2_−*M*_1_ (*M*_2_ is the endpoint mean, *M*_1_ is the baseline mean); $SD=\sqrt{SD_{1}^{2}+SD_{2}^{2}-\left(2\times \mathrm{Corr}\times SD_{1}\times SD_{2}\right)}$ (SD_1_ is the baseline SD, SD_2_ is the endpoint SD), Corr is the correlation coefficient between the baseline and endpoint scores, conservatively set at 0.5 (Follmann et al., 1992; Fukuta et al., 2016). Effect sizes are expressed using Hedges’ g (g) and 95% Cl. g < 0.2 indicates a small effect, 0.2 < g < 0.8 indicates a medium effect, and g > 0.8 indicates a significant effect (Jacob, 1998). *I*^2^ < 50% uses the fixed-effects model; *I*^2^ > 50% uses the random-effects model to combine effect sizes, then conduct sensitivity and subgroup analyses (Higgins and Thompson, 2002). *p* < 0.05 was defined as statistically significant (Higgins et al., 2011).

## 3 Results

### 3.1 Study selection and characteristics

**Figure 1:**
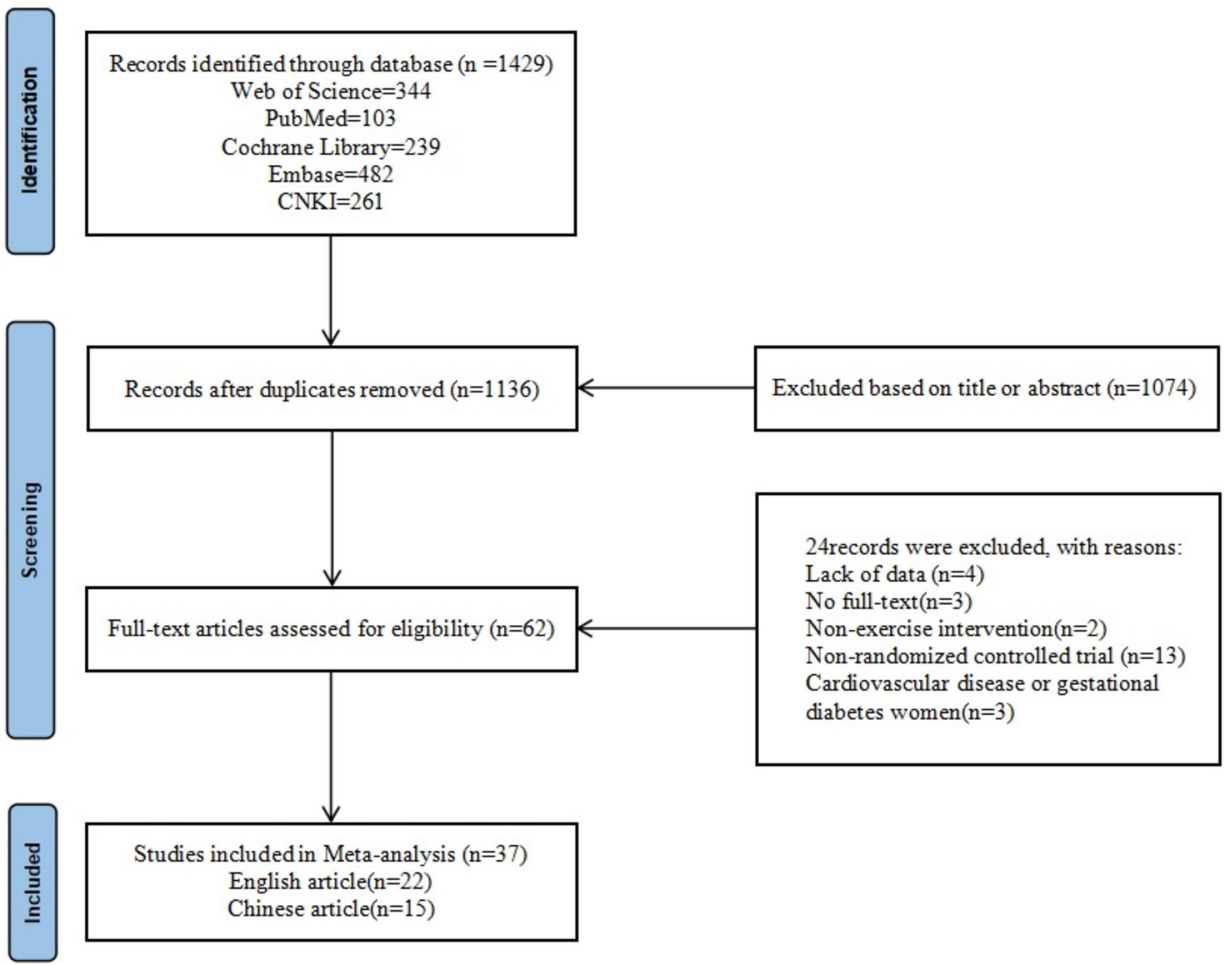
Literature screening flowchart.

A total of 1,429 literatures were retrieved, 1,136 literature remained after eliminating duplicates, and 62 literatures were obtained by further screening based on title and abstract. Finally, 24 papers were excluded due to missing data, unavailability of full text, non-exercise intervention, non-RCT, and disease. A total of 37 papers were finally included, including 22 English papers and 15 Chinese papers (Figure 1).

### 3.2 Results of the quality assessment of the included literature

**Figure 2:**
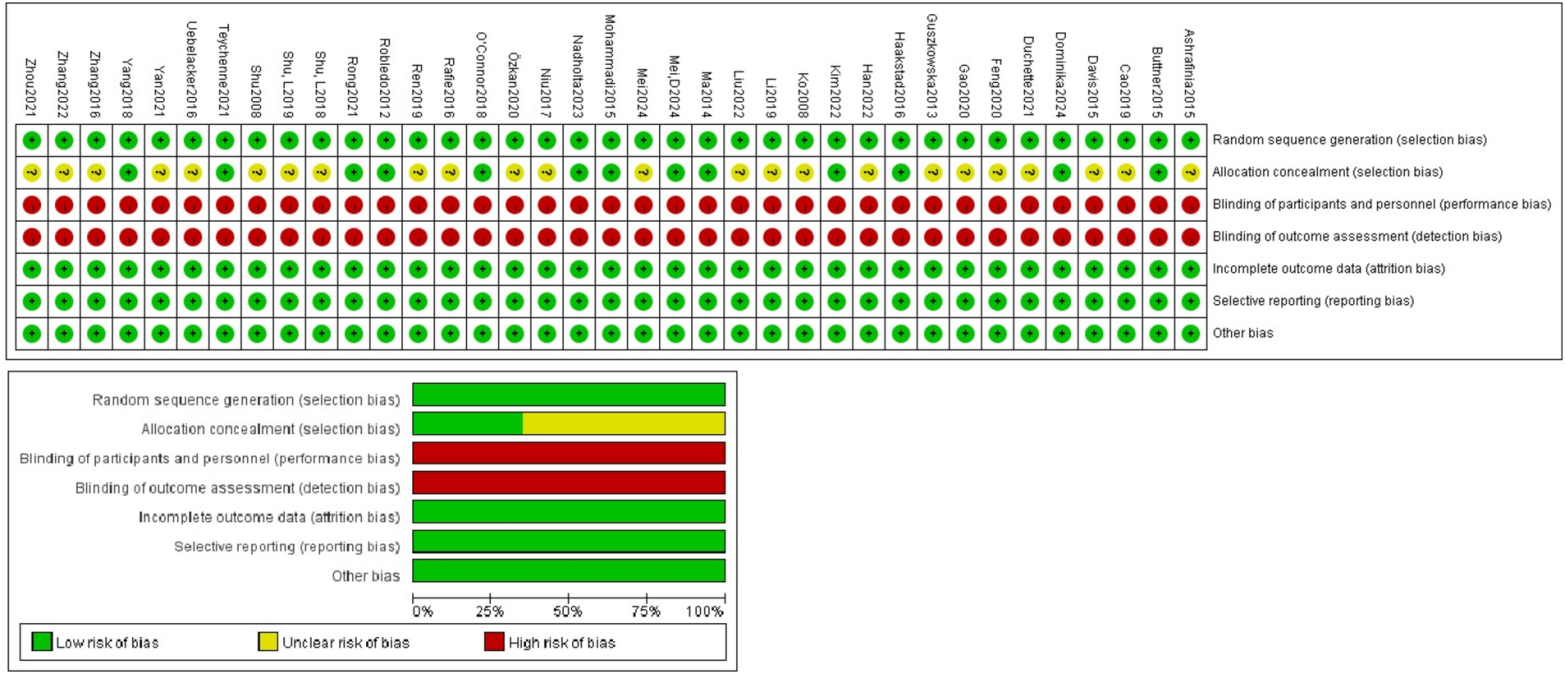
Literature quality evaluation.

The 37 included literature all used randomized methods to allocate members of the experimental and control groups, provided complete data, reported results unselectively, and found no other bias. 13 literature described procedures for allocating concealment, and all literature were not blinded to the subjects and implementers (Figure 2). The quality of the included literatures was assessed using the PEDro scale. The average quality of the included literatures was 7.35, all included literatures demonstrated high methodological quality and there were no low-quality literatures (Supplementary material).

### 3.3 Characteristics of the included studies

There were 37 RCT. Thirty literatures included depression as an outcome indicator, and a total of 7 different measure tools were used, including the Edinburgh Postnatal Depression Scale (EPDS). Seventeen literatures included anxiety as an outcome indicator, and a total of 8 different measure tools were used, including the Hamilton Anxiety Rating Scale (HARS). Nine literatures included fatigue as an outcome indicator, and a total of seven different measure tools were used, including the Fatigue Assessment Instrument (FAI). The exercise content included yoga, aerobic exercise, etc.; the single exercise time ranged from 15 to 90 min; the frequency ranged from 1–5 times/week; the exercise cycle ranged from 4 to 16 weeks; the exercise intensities were mainly low-intensity, moderate-intensity, and low-moderate-intensity; the exercise formats included group format, individual format, and group + individual format; and the intervention Period includes prenatal intervention, postpartum intervention, and prenatal + postpartum intervention. In the control group, routine care and daily activities were used. The basic characteristics of the included literature are shown in Table 1.

**Table 1:** Characteristics of the included literature.

First author	Country	Sample size (experimental group/control group)	Target sample	Intervention period	Intervention in the experimental group (exercise format)	Intervention in the control group	Exercise intensity	Frequency	Time	Exercise cycle	Measure tools
Ashrafinia (2015)	Iranian	80 (40/40)	Healthy postnatal women	Postnatal interventions (72 h after childbirth)	Pilates (individual exercises at home)	Non-participation	Low-Medium	5 times/week	30 min/time	8 weeks	MFI-20
Buttner (2015)	USA	57 (28/29)	Women with postnatal depression (within 12 months of delivery)	Postnatal interventions	Yoga (group exercise)	Non-participation	Low	2 times/week	60 min/time	8 weeks	HDRS, IDAS
Ca (2019)	China	128 (65/63)	Healthy pregnant women	Intervention at 1 weeks postpartum	Pelvic floor muscle rehabilitation exercises (individual exercises)	Routine care	Medium	3 times/day	20 min/time	14 weeks	EPDS, SAS
Davis (2015)	USA	46 (23/23)	Pregnant women with depression or anxiety (≤28 weeks of pregnancy)	Prenatal intervention	Yoga (group exercise)	Conventional treatment	Low	1 time/week	75 min/time	8 weeks	EPDS, STAI
Dominika (2024)	Polish	54 (34/20)	Healthy pregnant women (18–26 weeks of pregnancy)	Prenatal intervention	HIIT (group exercises)	Healthy lifestyle education	82–92% predicted heart rate	3 times/week	60 min/time	8 weeks	BDI-II
Duchette (2021)	USA	19(10/9)	Healthy pregnant women (12–26 weeks of pregnancy)	Prenatal intervention	Yoga (individual exercise at home)	Routine care	Low	1 time/week	90 min/time	10 weeks	POMS
Feng (2020)	China	100 (50/50)	Healthy pregnant women (28–36 weeks of pregnancy)	Prenatal intervention	Positive thinking yoga (Group exercise + Individual exercises at home)	Routine care	Low	Group practice: 1 time/week; Individual practice at home: 2 times/day	Group exercise: 60 min/time; Individual exercise at home: 20 min/time	4 weeks	SAS
Gao (2020)	China	84 (42/42)	Women with postnatal depression	Postnatal interventions	Yoga exercise (group exercise)	Routine care	Low-medium	4 times/week	60 min/time	6 weeks	HDRS, HARS
Guszkowska (2013)	Polish	97 (56/41)	Healthy pregnant women (20–34 weeks of pregnancy)	Prenatal intervention	Aerobic exercise (group exercise)	Routine care	Low-medium	2 times/week	50 min/time	8 weeks	POMS
Haakstad (2016)	Norway	105 (52/53)	Healthy pregnant women (12–24 weeks of pregnancy)	Prenatal intervention	Aerobic dance (group exercises)	Daily activities	Low-medium	2 times/week	60 min/time	12 weeks	WHOQOL, SF-36
Han (2022)	China	70 (35/35)	Women with postnatal depression	Postnatal interventions	Aerobic exercise (group exercise)	Cognitive therapy	60–80% predicted heart rate	3 times/week	40 min/time	12 weeks	EPDS
Kim (2022)	Korea	16 (8/8)	Healthy pregnant women under 40 years of age (24–28 weeks of pregnancy)	Prenatal intervention	Pilates (individual exercises at home)	Non-participation	50–60% predicted heart rate	2 times/week	50 min/time	8 weeks	EPDS
Ko (2008)	China-Taiwan	61 (31/30)	Healthy Postnatal Women	Prenatal intervention	Pilates (group practice)	Routine care	50–60% predicted heart rate	3 times/week	60 min/time	8 weeks	FSC, CES-D
Li (2019)	China	100 (50/50)	Women with postnatal depression	Postnatal interventions	Yoga exercise (group exercise)	Routine care + Psychological counseling	Low-medium	2 times/week	60 min/time	8 weeks	EPDS, SAS
Liu (2022)	China	86 (43/43)	Healthy Second-Trimester Pregnant Women	Postnatal interventions	Positive thinking yoga (Individual exercises at home)	Psychological counseling	Low-medium	2 times/week	30 min/time	8 weeks	EPDS, HAD
Ma (2014)	China	82 (40/42)	Women with postnatal depression	Postnatal interventions	Yoga exercise (group exercise)	Routine care	Low	4 times/week	60 min/time	8 weeks	EPDS, SAS
Mei (2024)	China	60 (30/30)	Healthy pregnant women (week of pregnancy ≤28 weeks)	Prenatal intervention	Aerobic exercise (yoga, tai chi) + resistance exercise (group exercises)	Routine antenatal education	Low	3 times/week	30 min/time	6 weeks	HARS
Mei, D (2024)	China	125 (63/62)	Women with postnatal depression	Postnatal interventions	Aerobic exercise (individual exercises)	Non-participation	Medium	1 time/3 days	30-40 min/time	12 weeks	EPDS
Mohammadi (2015)	Iranian	85 (43/42)	Healthy pregnant women (26–32 weeks of pregnancy)	Prenatal intervention + Postnatal interventions	Stretching and breathing exercises (individual exercises at home)	Routine prenatal and postnatal education	Medium	3 times/week	20-30 min/time	Until 8 weeks postpartum	EPDS, FIF
Nadholta (2023)	India	77 (34/43)	Healthy pregnant women	Prenatal intervention	Yoga (individual online exercises)	Routine care	Low	5 times/week	40-60 min/time	16 weeks	DASS-42
Niu (2017)	China	80 (40/40)	Women with postnatal depression	Intervention at 1 weeks postpartum	Yoga (Individual exercises at home)	Routine care	Low-medium	1 time/week	60 min/time	12 weeks	HDRS, HARS
OʼConnor (2018)	USA	89 (44/45)	Healthy pregnant women (21–25 weeks of pregnancy)	Prenatal intervention	Resistance exercises + Aerobic exercise (group exercises)	Routine care	Low-medium	2 times/week	17 min/time	12 weeks	POMS
Özkan (2020)	Istanbul	65 (34/31)	Women with post-natal depression (age 25–35)	Postnatal interventions	Physical exercise (individual exercises)	Non-participation	Medium	5 times/week	30 min/time	4 weeks	EPDS
Rafie (2016)	Egypt	100 (50/50)	Healthy pregnant women (16–27 weeks of pregnancy)	Prenatal intervention	Aerobic exercise (group exercise)	Routine care	Medium	3 times/week	60 min/time	12 weeks	CES-D
Ren (2019)	China	38 (19/19)	Women with postnatal depression	Intervention at 6 weeks postpartum	Aerobic exercise (group exercise)	Daily activities	Medium	3 times/week	40 min/time	12 weeks	EPDS
Robledo (2012)	Colombia	74 (37/37)	Healthy pregnant women (16–20 weeks of pregnancy)	Prenatal intervention	Walking + aerobic exercise + stretching + relaxation (group exercise)	Daily activities	Medium	3 times/week	60 min/time	12 weeks	CES-D
Rong (2021)	China	64 (32/32)	Healthy pregnant women (18–27 weeks of pregnancy)	Prenatal intervention	Yoga exercise (group exercise)	Routine care	Low	3 times/week	60 min/time	12 weeks	EPDS, S-AI
Shu (2008)	China-Taiwan	68 (35/33)	Women with post-natal depression (age 20–35)	Intervention at 4 weeks postpartum	Stretching + relaxation exercises (group exercises + individual exercises at home)	Non-participation	Medium	Group exercises: 1 time/week; Individual exercises at home: 2 times/week	60 min/time	12 weeks	EPDS
Shu, L (2018)	China	115 (55/60)	Pregnant women with anxiety disorders (10–32 weeks of pregnancy)	Prenatal intervention	Positive Thinking Yoga (Group exercise + Individual exercise at home)	Psychological counseling	Low	Group practice: 1 time/week; Individual practice at home: 2 times/day	Group exercise: 60 min/time; Individual exercise at home: 20 min/time	4 weeks	SAS
Shu, L (2019)	China	86 (43/43)	Pregnant women with anxiety or depression (10–28 weeks of gestation)	Prenatal intervention	Group Positive Thinking Yoga (group exercise)	Psychological counseling	Low	1 time/week	60 min/time	6 weeks	EPDS, SAS
Teychenne (2021)	Australia	62 (32/30)	Women with postnatal depression (3–9 months postnatal)	Postnatal interventions	Family physical activity (Individual exercise at home)	Daily activities	Medium	Self-organization	Self-organization	12 weeks	EPDS
Uebelacker (2016)	USA	20 (12/8)	Pregnant women with depression (12–26 weeks of gestation)	Prenatal intervention	Yoga (group exercise + individual practice at home)	Mom-Baby Wellness Workshop (MBWW)	Low	2 times/week	75 min/time	9 weeks	EPDS
Yan (2021)	China	246 (123/123)	Healthy pregnant women (28 weeks of pregnancy and above, age 35–40)	Prenatal intervention	Fertility dance exercise (group exercise + individual exercise)	Routine care	Low-Medium	1 time/week	90 min/time	Until 6 weeks postpartum	EPDS
Yang (2018)	China-Taiwan	129 (64/65)	Healthy Postnatal Women	Intervention at 6 weeks postpartum	Aerobic gymnastics (individual exercises)	Routine care	Low	3 times/week	15 min/time	12 weeks	PFS, EPDS
Zhang (2016)	China	164 (82/82)	Healthy pregnant women	prenatal intervention	Yoga exercise (group exercise)	Routine care	Low	2 times/week	75 min/time	8 weeks	SDS, SAS
Zhang (2022)	China	209 (109/100)	Healthy pregnant women (24–27 weeks of pregnancy)	Prenatal intervention	Childbirth ball exercises based on abdominal core training (individual exercises)	Routine care	Medium	4 times/week	30 min/time	8 weeks	FAI
Zhou (2021)	China	90 (45/45)	Pregnant women with anxiety or depression (26–29 weeks gestation)	Prenatal intervention	Positive thinking yoga (Group exercise + Individual exercise at home)	Pregnancy education + psychological interventions	Low	Group exercise: 1 time/week; Individual exercise at home: 1 time/day	Group exercises: 90 min/time; Individual exercises at home: 30 min/time	6 weeks	SDS, SAS

BDI-II, Beck depression inventory—II; CES-D, Center for Epidemiologic Studies Depression Scale; DASS-42, Depression Anxiety Stress Scale; EPDS, Edinburgh Postnatal Depression Scale; FIF, Fatigue Identification Form; FAI, Fatigue Assessment Instrument; FSC, Fatigue Symptom Checklist; HAD, Hospital Anxiety Depression Scale; HDRS, Hamilton Depression Rating Scale; HARS, Hamilton Anxiety Rating Scale; IDAS, Inventory of Depression and Anxiety Symptoms; MFI-20, Multidimensional Fatigue Inventory; PFS, Postpartum Fatigue Scale; POMS, Profile of Mood States Questionnaire; S-AI, State Anxiety Inventory; SAS, Self-rating Anxiety Scale; SDS, Self-rating Depression Scale; STAI, State–trait anxiety inventory; WHOQOL, World Health Organization Quality of Life-BREF scale; SF-36, the 36-item Short Form Health Survey.

### 3.4 Test for publication bias

**Figure 3:**
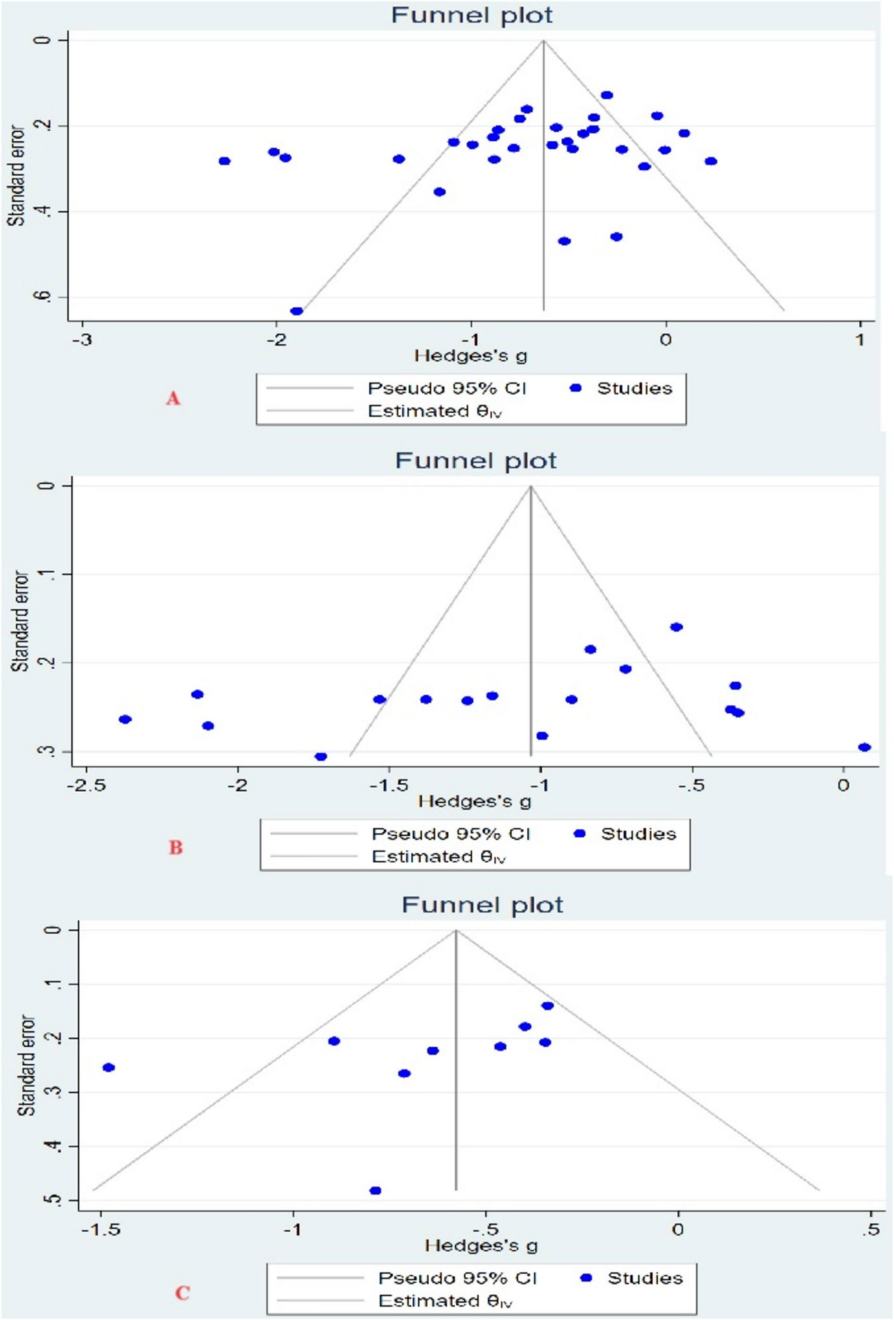
Funnel plot. (A) Funnel plot of depression indicators; (B) funnel plot of anxiety indicators; (C) funnel plot of fatigue indicators.

Using Egger’s test, Begg’s test, and funnel plot to test for the presence of publication bias. There was no significant asymmetry in the 3 funnel plots (Figure 3). For the depression indicator, Egger’s test (*Z* = −1.51, *p* = 0.13) and Begg’s test (*Z* = −1.78, *p* = 0.08); for the anxiety indicator, Egger’s test (*Z* = −0.91, *p* = 0.36) and Begg’s test (*Z* = −0.87, *p* = 0.43); for the fatigue indicator, Egger’s test (*Z* = −1.39, *p* = 0.16), Begg’s test (*Z* = −1.98, *p* = 0.08). It showed that there was no significant publication bias among the studies for the 3 indicators.

### 3.5 Sensitivity analysis

**Figure 4:**
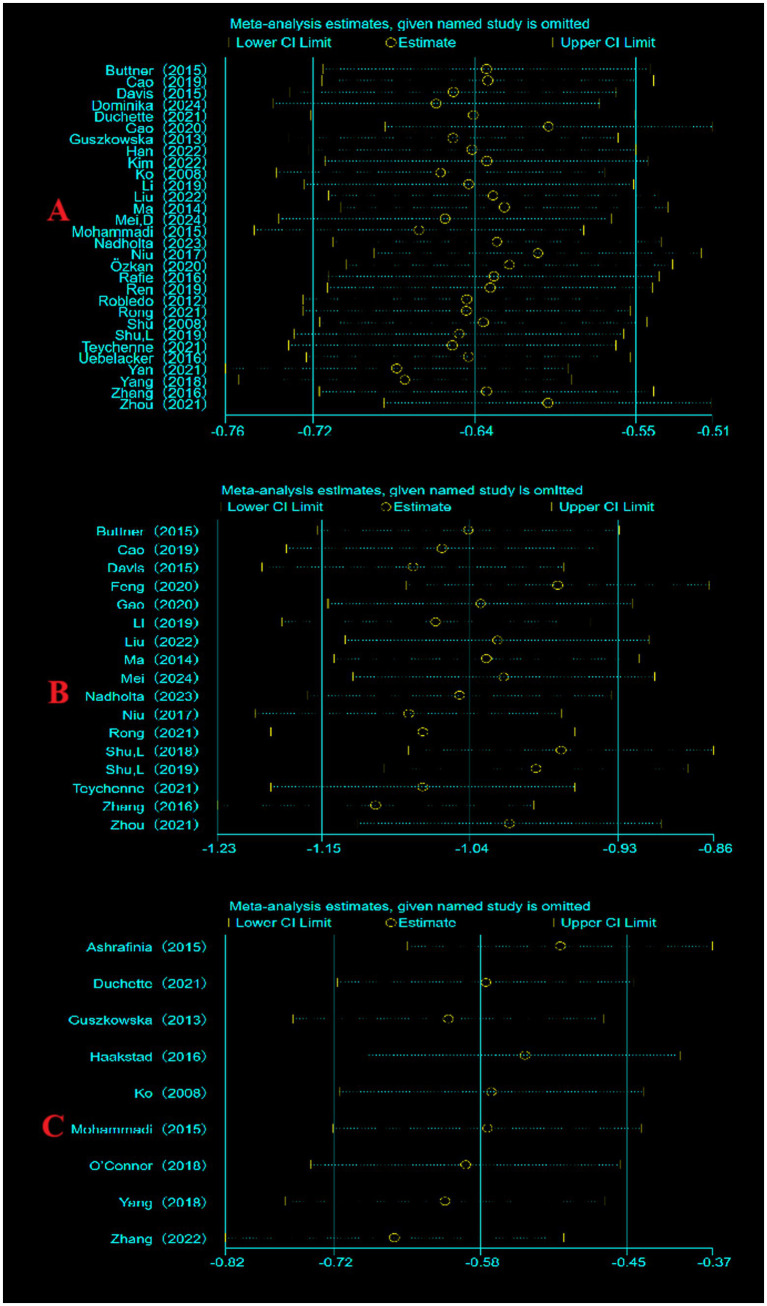
Sensitivity analysis. (A) Sensitivity analysis of depression indicators; (B) sensitivity analysis of anxiety indicators; (C) sensitivity analysis of fatigue indicators.

Sensitivity analyses were performed for each of the 3 indicators included literature, to evaluate the reliability by excluding literature one by one. No significant differences were found in the results, indicating that the results of this meta-analysis are credible (Figure 4).

### 3.6 Meta-analysis results

#### 3.6.1 Depression indicators

**Figure 5:**
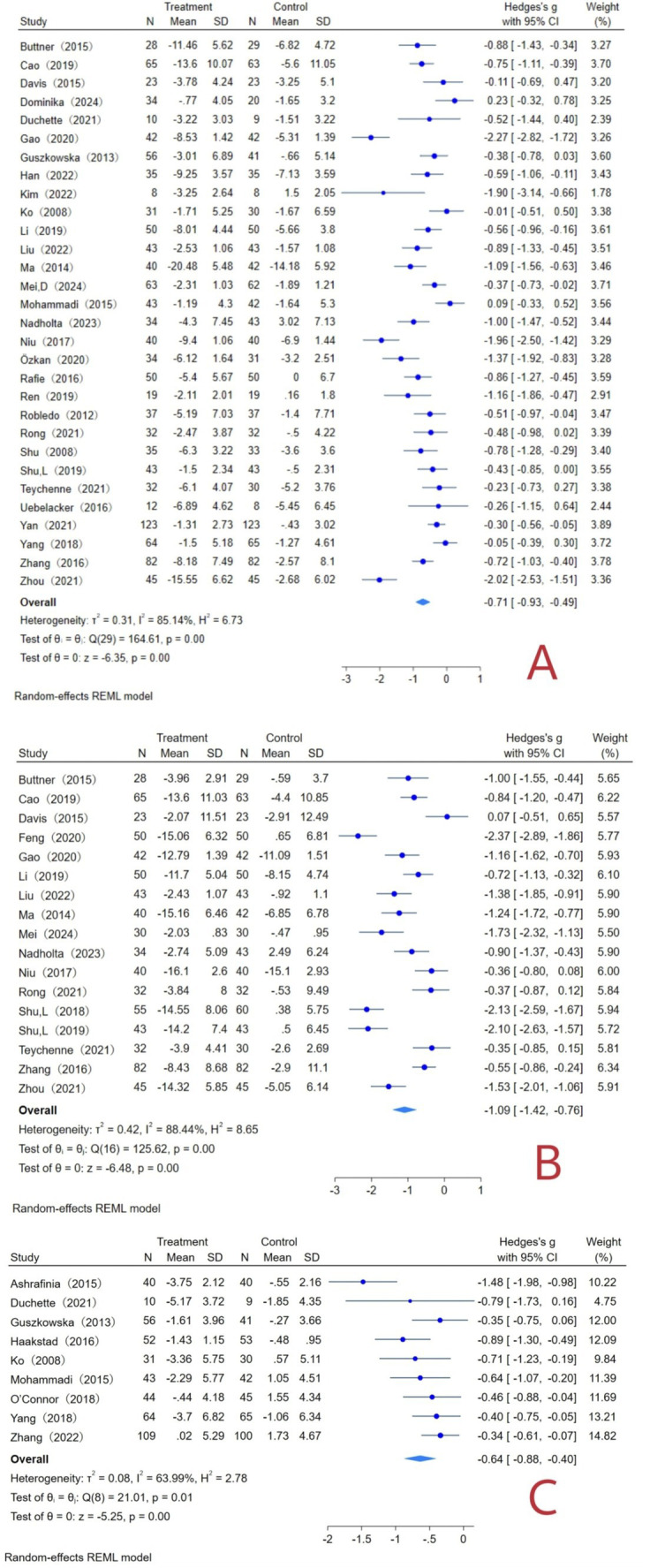
Exercise interventions and maternal forest plot. (A) Forest plot of depression indicators; (B) forest plot of anxiety indicators; (C) forest plot of fatigue indicator.

A total of 30 literatures with 2,473 subjects were included, with a total sample size of 1,253 in the experimental group and 1,220 in the control group. The overall heterogeneity test (*I*^2^ = 85.14%, *p* = 0.00) indicated that there was heterogeneity among multiple studies, so using the random effects model to combined effect sizes: [*g* = −0.71, 95% CI (−0.93, −0.49), *p* = 0.00], was statistically significant (Figure 5A). It showed that exercise can improve maternal depression symptoms with a medium effect.

#### 3.6.2 Anxiety indicators

A total of 17 literatures with 1,481 subjects were included, with a total sample size of 734 cases in the experimental group and 747 cases in the control group. The overall heterogeneity test (*I*^2^ = 88.44%, *p* = 0.00) indicated that there was heterogeneity among multiple studies, so using the random effects model to combine the effect sizes: [*g* = −1.09, 95% CI (−1.42, −0.76), *p* = 0.00], was statistically significant (Figure 5B). It showed that exercise can improve maternal anxiety symptoms with a significant effect.

#### 3.6.3 Fatigue indicators

A total of 9 literatures with 874 subjects were included, with a total sample size of 449 cases in the experimental group and 425 cases in the control group. The overall heterogeneity test (*I*^2^ = 63.99%, *p* = 0.01) indicated that there was heterogeneity among multiple studies, so using the random effects model to combine the effect sizes: [*g* = −0.64, 95% CI (−0.88, −0.40), *p* = 0.00], was statistically significant (Figure 5C). It showed that exercise can improve maternal fatigue symptoms with a medium effect.

### 3.7 Subgroup analysis of moderators

In this study, exercises content, single exercise time, exercise frequency, exercise cycle, exercise intensity, exercise format and intervention period were set subgroups, respectively, analyzed (Table 2). To explore the sources of heterogeneity and to propose the best exercise intervention program.

**Table 2:** Analysis of moderating variables.

Moderators	Outcome	Homogeneity test			Category	Number of literatures	Number of samples	Effect size and 95% CI	Two-tailed test	
		*Q*	*p*	*I*^2^ (%)					*Q*	*p*
Exercise content	Depression	164.61	0.00	85.14	Yoga	13	1,035	−1.00 (−1.36, −0.63)	76.37	0.00
					Combination of aerobic exercise	15	1,361	−0.47 (−0.68, −0.26)	43.62	0.00
					Pilates	2	77	−0.87 (−2.71, 0.98)	7.70	0.359
	Anxiety	125.62	0.00	88.44	Yoga	14	1,231	−1.12 (−1.50, −0.75)	111.89	0.00
					Combination of aerobic exercise	3	250	−0.95 (−1.71, −0.19)	12.03	0.014
	Fatigue	21.01	0.007	63.99	Yoga	1	19	−0.79 (−1.73,0.16)	0.00	0.102
					Combination of aerobic exercise	6	714	−0.49 (−0.66, −0.32)	6.23	0.00
					Pilates	2	141	−1.10 (−1.85, −0.35)	4.39	0.004
Single exercise time	Depression	131.72	0.00	83.20	10–30 min/time	5	493	−0.58 (−1.10, −0.05)	28.83	0.031
					40–60 min/time	17	1,148	−0.82 (−1.13, −0.52)	86.07	0.00
					75–90 min/time	5	495	−0.42 (−0.68, −0.15)	5.45	0.002
	Anxiety	60.69	0.00	83.01	10-30 min/time	3	274	−0.97 (−1.35, −0.59)	7.35	0.00
					40–60 min/time	8	630	−1.27 (−1.79, −0.76)	33.66	0.00
					75–90 min/time	2	210	−0.29 (−0.89,0.31)	3.43	0.342
	Fatigue	21.01	0.007	63.99	10–30 min/time	5	592	−0.64 (−1.02, −0.25)	16.74	0.001
					40–60 min/time	3	263	−0.65 (−0.99, −0.30)	3.63	0.00
					75–90 min/time	1	19	−0.79 (−1.73,0.16)	0.00	0.102
Exercise frequency	Depression	131.28	0.00	83.98	1–2 times/week	12	1,052	−0.69 (−0.99, −0.39)	42.41	0.00
					3 times/week	10	743	−0.40 (−0.66, −0.13)	30.64	0.004
					4–5 times/week	4	308	−1.42 (−1.98, −0.86)	13.87	0.00
	Anxiety	60.69	0.00	83.01	1–2 times/week	7	619	−0.86 (−1.37, −0.34)	44.35	0.001
					3 times/week	3	252	−0.96 (−1.71, −0.21)	11.78	0.012
					4–5 times/week	3	243	−1.10 (−1.37, −0.83)	1.11	0.00
	Fatigue	21.01	0.007	63.99	1–2 times/week	4	310	−0.59 (−0.89, −0.29)	4.11	0.00
					3 times/week	3	275	−0.54 (−0.78, −0.30)	1.25	0.00
					4–5 times/week	2	289	−0.89 (−2.01,0.23)	15.50	0.118
Exercise cycle	Depression	144.63	0.00	83.78	4–8 weeks	14	903	−0.85 (−1.24, −0.46)	94.87	0.00
					9–12 weeks	12	849	−0.64 (−0.94, −0.35)	43.65	0.00
					14–16 weeks	2	205	−0.84 (−1.13, −0.55)	0.64	0.00
	Anxiety	125.62	0.00	88.44	4–8 weeks	6	1,070	−1.32 (−1.72, −0.92)	92.86	0.00
					9–12 weeks	6	206	−0.36 (−0.64, −0.08)	0.00	0.011
					14–16 weeks	2	205	−0.86 (−1.15, −0.57)	0.04	0.00
	Fatigue	20.93	0.00	69.04	4–8 weeks	4	447	−0.70 (−1.21, −0.18)	17.03	0.008
					9–12 weeks	4	342	−0.59 (−0.87, −0.32)	3.88	0.00
Exercise intensity	Depression	164.61	0.00	85.14	Low	13	911	−0.69 (−1.03, −0.36)	61.36	0.00
					Medium	7	511	−0.63 (−1.00, −0.25)	25.24	0.001
					Low-medium	6	693	−1.04 (−1.71, −0.37)	64.86	0.002
					Medium-high	4	298	−0.46 (−0.90, −0.02)	9.80	0.041
	Anxiety	125.62	0.00	88.44	Low	11	941	−1.26 (−1.72, −0.79)	99.37	0.00
					Medium	2	190	−0.63 (−1.10, −0.15)	2.38	0.01
					Low-medium	4	350	−0.90 (−1.34, −0.45)	11.66	0.00
	Fatigue	21.01	0.007	63.99	Low	4	289	−0.83 (−1.34, −0.33)	12.22	0.001
					Medium	1	85	−0.64 (−1.08, −0.20)	0.00	0.004
					Low-medium	4	500	−0.49 (−0.75, −0.24)	5.51	0.00
Exercise format	Depression	164.61	0.00	85.14	Group	15	1,117	−0.65 (−0.93, −0.37)	63.55	0.00
					Individual	11	872	−0.76 (−1.15, −0.37)	65.28	0.00
					Group + individual	4	424	−0.86 (−1.66, −0.05)	35.70	0.039
	Anxiety	125.62	0.00	88.44	Group	9	743	−0.97 (−1.40, −0.54)	50.92	0.00
					Individual	5	433	−0.77 (−1.13, −0.40)	12.85	0.00
					Group + individual	3	305	−2.01 (−2.49, −1.52)	6.09	0.00
	Fatigue	21.01	0.007	63.99	Group	4	352	−0.6(−1.12, −0.27)	16.80	0.00
					Individual	5	522	−0.60 (−0.86, −0.34)	4.15	0.001
Intervention period	Depression	164.61	0.00	85.14	Prenatal	13	847	−0.66 (−0.98, −0.34)	52.03	0.00
					Postpartum	15	1,235	−0.85 (−1.17, −0.53)	91.73	0.00
					Prenatal+ Postpartum	2	331	−0.15 (−0.53,0.24)	2.48	0.458
	Anxiety	125.62	0.00	88.44	Prenatal	9	802	−0.88 (−1.14, −0.61)	18.39	0.00
					Postpartum	8	679	−1.29 (−1.86, −0.72)	98.85	0.00
	Fatigue	21.01	0.007	63.99	Prenatal	5	519	−0.51 (−0.75, −0.27)	5.92	0.00
					Postpartum	3	270	−0.85 (−1.48, −0.21)	12.21	0.009
					Prenatal+ Postpartum	1	85	−0.64 (−1.08, −0.20)	0.00	0.004

#### 3.7.1 Depression indicators

Subgroup analyses of the included literatures of depression indicators showed statistically significant maximum effect sizes in the subgroups of exercise content, single exercise time, exercise frequency, exercise cycle, exercise intensity, exercise format, and intervention period from: yoga (*g* = −1.00, *p* = 0.00), 40–60 min/time (*g* = −0.82, *p* = 0.00), 4–5 times/week (*g* = −1.42, *p* = 0.00), 4–8 weeks (*g* = −1.24, *p* = 0.00), low-medium intensity (*g* = −1.04, *p* = 0.002), group + individual format (*g* = −0.86, *p* = 0.039), postpartum intervention (*g* = −0.85, *p* = 0.00). Therefore, exercise intervention in the postpartum period, low-moderate intensity yoga with group + individual, 4–5 times/week, 40–60 min/time, duration 4–8 weeks may achieve the best effect in improving maternal depressive symptoms.

#### 3.7.2 Anxiety indicators

Subgroup analyses of the included literatures of anxiety indicators showed statistically significant maximum effect sizes in the subgroups of exercise content, single exercise time, exercise frequency, exercise cycle, exercise intensity, exercise format, and intervention period from: yoga (*g* = −1.12, *p* = 0.00), 40–60 min/time (*g* = −1.27, *p* = 0.00), 4–5 times/week (*g* = −1.10, *p* = 0.00), 4–8 weeks (*g* = −1.32, *p* = 0.00), low intensity (*g* = −1.26, *p* = 0.00), group + individual format (*g* = −2.01, *p* = 0.00), postpartum intervention (*g* = −1.29, *p* = 0.00). Therefore, exercise intervention in the postpartum period, low intensity yoga with group + individual, 4–5 times/week, 40–60 min/time, duration 4–8 weeks may achieve the best effect in improving maternal anxiety symptoms.

#### 3.7.3 Fatigue indicators

Subgroup analyses of the included literatures of fatigue indicators showed statistically significant maximum effect sizes in the subgroups of exercise content, single exercise time, exercise frequency, exercise cycle, exercise intensity, exercise format, and intervention period from: Pilates (*g* = −1.10, *p* = 0.004), 40–60 min/time (*g* = −0.65, *p* = 0.00), 1–2 times/week (*g* = −0.59, *p* = 0.00), 4–8 weeks (*g* = −0.70, *p* = 0.008), low intensity (*g* = −0.83, *p* = 0.001), group format (*g* = −0.69, *p* = 0.00), postpartum intervention (*g* = −0.85, *p* = 0.009). Therefore, exercise intervention in the postpartum period, low intensity yoga with group, 1–2 times/week, 40–60 min/time, duration 4–8 weeks may achieve the best effect in improving maternal fatigue symptoms.

## 4 Discussion

### 4.1 Quality assessment of the included literature

There was no harmonized measure tools used in the literature included for the 3 indicators, with a total of 7 different tools used for the depression indicator, 8 different tools used for the anxiety indicator, and 7 different tools used for the fatigue indicator. The lack of harmonized measure tools to assess maternal symptoms of depression, anxiety, and fatigue may be one of the reasons for the high level of heterogeneity.

None of the included literature was blinded to the subjects and experiment conductors, which affected the quality of the literature, but did not affect the effectiveness of the exercise intervention on maternal depression, anxiety and fatigue. Because of the specificity of the maternal population and the nature of the exercise intervention, it was beneficial for subjects and implementers to understand the purpose of the experiment. Therefore, not blinding subjects and implementers did not affect the results of the experiment.

### 4.2 Analysis of overall effect

The results of our study are consistent with previous research (Cai et al., 2022; Ji et al., 2024; Liu et al., 2020), that maternal can benefit from exercise and significant improvements in depression, anxiety and fatigue. Research suggests that exercise may be a potential alternative to antidepressant and anxiolytic medications and has a proximate effect to pharmacologic interventions in alleviating depression and anxiety symptoms (Guerrera et al., 2020; Fox et al., 2023). However, as pharmacologic interventions may cause some degree of maternal side effects (Marasine et al., 2020), women are reluctant to take medications, resulting in low compliance (Zhao et al., 2020). Therefore, exercise may be a safer and more effective intervention to improve maternal depression and anxiety. Exercise improves maternal depression, anxiety, and fatigue in 3 main ways: social support during exercise, positive experiences of exercise, and physiologic changes in the body produced by exercise (Jia et al., 2018). First, in terms of physiological regulation, exercise can elevate the level of neurotrophic factors in the body, improve neurotransmitter expression and other endocrine regulatory mechanisms, thus intervening maternal depression and anxiety (Li and Gao, 2019). Furthermore, it can treat depression, anxiety and mental fatigue by remodeling brain structure, activating brain targets, maintaining the volume of the prefrontal cortex and hippocampus, improving mood and releasing psychological stress (Thomas et al., 2016; Maass et al., 2015). Secondly, body shape changes, weakened mobility and lifestyle variations are important causes of maternal depression, anxiety and fatigue (Badr and Zauszniewski, 2017; Doering Runquist et al., 2009). Exercise can promote the recovery of maternal body functions and image, regulate the psychological state, and quickly adapt to the change of their own social roles, which in turn can effectively improve maternal depression and anxiety (Bershadsky et al., 2014). The recovery of physical function can help to improve maternal anti-fatigue ability and improve fatigue symptoms (Liu et al., 2020). Compared to cognitive-behavioral treatments and other interventions, exercise interventions have a strong potential advantage (van Lieshout et al., 2021), with social support and positive experiences for maternal in exercise and are more accessible and cost-effective for women who have low income, learning comprehension difficulties, and lower levels of education (Hedman-Lagerlöf et al., 2021). Despite the many benefits of exercise intervention, it is not suitable for all maternal. Exercise is not suitable for women with gestational hypertension, placenta previa, and conditions such as a weakened thyroid gland, or women who have delivered by cesarean section for less than 6 weeks (Corwin and Arbour, 2007; Hinman et al., 2015). Therefore, before exercise begins, clinicians should assess maternal fitness to ensure the safety of the exercise intervention.

### 4.3 Exercise content

In the depression and anxiety indicators, the results of the subgroup analyses of exercise content were consistent, yoga had the best intervention effect. In the fatigue indicator, Pilates had the best intervention effect. Yoga and Pilates are popular in the maternal population and have high reliability and satisfaction in improving maternal depression, anxiety, and fatigue (Nadholta et al., 2023; Ashrafinia et al., 2015). Yoga and Pilates mainly consist of breathing exercises, body posture adjustments, meditation, stretching, and relaxation exercises (Rong et al., 2021). Yoga can reduce depression, anxiety, and labor pain throughout pregnancy, enhance immunity, and improve mental health (Field et al., 2012). Meditation practice in yoga can improve maternal concentration, exclude the interference of the external environment, make their mood calmer and more relaxed (Campbell and Nolan, 2016), improve the sense of self-efficacy of pregnant women in childbirth, enhance confidence in childbirth, and help to alleviate the depression, anxiety and physiological discomfort of childbirth (Tilden et al., 2016). For postpartum women, yoga can help them improve their physical form and achieve improvement in postpartum depression and anxiety (Li and Gao, 2019). Pilates has a positive impact on physical and mental health and is considered an effective measure for intervention and treatment of maternal fatigue (Ko et al., 2008). The stretching exercises in Pilates help to improve maternal flexibility and joint flexibility, which play a role in improving self-care, increasing the level of daily activities and relieving low back pain, which achieves the effect of relieving the symptoms of maternal fatigue (Ashrafinia et al., 2015).

### 4.4 Time, frequency, cycle, intensity and format of exercise

Our study showed that subgroup analyses of the 3 indicators regarding single exercise time and exercise cycle were consistent, 40–60 min/time, 4–8 weeks have the best intervention effect. Shorter exercise time may limit accurate assessment and fail to ensure long-term gains. Longer time can aggravate the physical and psychological burden on the maternal body and even trigger a range of inferiority (Dipietro et al., 2019). In addition, the American College of Obstetricians and Gynecologists (ACOG) recommends that single exercise time of 30 min or more and more than 150 min per week are more effective, which is consistent with our study results (Feng et al., 2019). During pregnancy, prolonged exercise may lead to miscarriage due to embryonic instability and other reasons; after delivery, excessive exercise may cause harm to the maternal body due to temporary mobility problems (Liu et al., 2023). Therefore, longer cycle exercise interventions may not be appropriate for maternal. Research pointed out that the exercise cycle of 4-8 weeks can not only achieve a good intervention effect, but also enable pregnant women to develop the habit of exercise, which is a more appropriate exercise cycle (Liu et al., 2020).

Exercise frequency is an important factor influencing the effectiveness of the intervention. The results of this study showed that 4–5 times/week was the best intervention for depression and anxiety. 1–2 times/week was the best intervention for fatigue. Maternal depression and anxiety symptoms declined with increased exercise frequency, and there was a negative correlation between them (Jia et al., 2021). Furthermore, the recommendations of ACOG provide support for this research’s findings (Feng et al., 2019). The subgroup analysis of fatigue indicators was inconsistent, probably because low-frequency exercise is more conducive to relieving maternal low back pain, relaxing the body and mind, and does not aggravate physical and mental stress and burden, which is more effective in improving maternal fatigue (Liu et al., 2024).

Exercise intensity plays an important role in improving depression, anxiety, and fatigue symptoms, but information on best exercise intensity is still limited and needs to be further explored. The results of this study showed that Low intensity was the best intervention for anxiety and fatigue, and Low-medium intensity was the best intervention for depression. Although the results were inconsistent, they further validated the effectiveness of Low intensity. For maternal, exercise intensity should be Low intensity or Low-medium intensity to avoid high intensity exercise causing harm to both the mother and the fetus (Sánchez-Polán et al., 2021). Research has shown that low or medium intensity aerobic exercise is a recognized exercise intensity that not only protects maternal cardiovascular function, but also does not exacerbate post-exercise fatigue (Cai et al., 2022; Dipietro et al., 2019).

In this study, group + individual format was the best intervention for depression and anxiety, and group format was the best intervention for fatigue. Group-based exercise can give full play to face-to-face supervision, which can enhance compliance, and the effectiveness of interventions compared with unsupervised exercise (Liu et al., 2019). In addition, group exercise can help maternal gain more social support from peers and coaches, which, along with professional guidance from coaches and supervision from clinicians, greatly enhances the safety of the exercise (Liu et al., 2023). But group exercise requires adequate space and a high level of organizational skills on the part of the organizer to coordinate the time and place of all participants. Individual exercise is not limited by time and place, which can enhance maternal subjectivity and freedom (Zhang et al., 2019). For healthcare organizations with limited resources, combining group and individual exercise may be a better choice, but movement instruction and safety preaching should be done before individual exercise.

### 4.5 Intervention period

The results of the subgroup analyses on the intervention period in the 3 indicators were consistent, postpartum intervention was the best effect. Pregnant women’s perception of exercise is more scarce, they believe that exercise during pregnancy is not safe, so they rarely exercise to avoid harm to the fetus (Liu et al., 2020). After childbirth, the sudden shift in women’s social identity, changes in body shape, and decreased sleep quality due to caring for the baby may lead to more severe symptoms of depression, anxiety, and fatigue than in the prenatal period (Ji et al., 2024; Iwata et al., 2018). Compared with exercise during pregnancy, postpartum intervention may be more effective (Li and Gao, 2019), and the fact that postpartum exercise is more safe and convenient and mothers no longer have to worry about the adverse effects of exercise on the fetus, it’s may be one of the reasons why postpartum intervention is better than prenatal intervention (Dipietro et al., 2019).

### 4.6 Comparison with previously published meta-analyses

Although this meta-analysis is consistent with the results of most of the existing literature that validate the effectiveness of exercise intervention on maternal depression, anxiety and fatigue. However, compared to other research, this review excluded combined interventions, only analyzed exercise intervention, and included all exercise types, which is more helpful in evaluating the final effect of exercise, and the results have a higher reliability. This review focuses on introducing refined subgroup analyses to dissect the effects of exercise content, single exercise time, exercise frequency, exercise cycle, exercise intensity, exercise format, and intervention period on maternal depression, anxiety, and fatigue, which have not been extensively explored in previous meta-analyses, and which may provide more specificity for the implementation or development of exercise intervention programs.

### 4.7 Limitations and perspectives

There are some limitations in our study, which we hope to further improve in future research and practice. First, although most trials have reported positive effects of exercise interventions on maternal depression, anxiety, and fatigue, the same exercise intervention program may have different effects on maternal women with different levels of depression, anxiety, and fatigue (e.g., mild, moderate, severe). Researchers should explore more refined and appropriate exercise intervention protocols based on different levels of maternal depression, anxiety, and fatigue to seek continuous optimization of intervention effects. Second, none of the subjects in the included literature had any diseases or comorbidities; therefore, the results of this study may not be appropriate for all maternal. Researchers should clearly define the maternal population that is suitable to participate in exercise, which could be effective in improving the safety of exercise intervention. For maternal who have diseases or complications but can still participate exercise, researchers should explore suitable exercise programs and pay more attention to exercise frequency, exercise cycle, and exercise intensity factors in the exercise program. Thirdly, while this study suggests the best exercise content, exercise intensity and exercise frequency, etc., these factors are key information for precise interventions. However, the current evidence in this area is not sufficient. Researchers should conduct more high-quality experiments to determine the best exercise content, exercise intensity and exercise frequency, etc., which can help to develop a more standardized exercise intervention program. Fourth, the included literature did not use a harmonized measurement tool, which may have been heterogeneous. Researchers should develop harmonized measurement tools to address the specificities of the maternal population to improve the accuracy of the assessment. Fifth, due to the specificity of the maternal population and the nature of the exercise intervention, blinding was not implemented during the experiment, which had a certain risk of bias and affected the quality assessment results to a certain extent. Researchers should strictly follow the guidelines for randomized controlled trials to ensure the reliability of the results.

## 5 Conclusions and recommendations

Our meta-analysis suggests that exercise can effectively improve maternal depression, anxiety and fatigue symptoms. Postnatal intervention may be more effective than prenatal intervention. Low-moderate intensity yoga with group + individual, 4–5 times/week, 40–60 min/time, duration 4–8 weeks is more effective in improving depressive symptoms; Low intensity yoga with group + individual, 4–5 times/week, 40–60 min/time, duration 4–8 weeks is more effective in improving anxiety symptoms; Low intensity Pilates with group, 1–2 times/week, 40–60 min/time, duration 4–8 weeks is more effective in improving fatigue symptoms. Despite the promising findings, there is less research on exercise intervention programs and more research is needed to support these findings.

Clinicians should consider factors such as exercise content, frequency and intensity when developing an exercise intervention program, and can use the results of this study as a reference. For example, it is more appropriate to choose light exercise such as yoga or Pilates, to take group exercise with low or moderate intensity, and to choose an intervention cycle of 4–8 weeks, 1–5 times/week, 40–60 min/time. Second, the group exercise movement process should ensure that the movements are standardized to avoid any other repercussions due to irregular movements. Choosing group + individual exercise may be a better option for healthcare organizations with limited resources, but it is important to provide movement instructions and safety presentations in advance. Finally, the monitoring of the exercise intervention process should be strengthened by measuring maternal heart rate and subjective perception at regular intervals during exercise to maximize the avoidance of exercise risks for maternal.

## Data Availability

The original contributions presented in the study are included in the article/Supplementary material, further inquiries can be directed to the corresponding authors.
